# HIV prevalence and factors associated with HIV infection among male injection drug users under 30: a cross-sectional study in Long An, Vietnam

**DOI:** 10.1186/1471-2458-6-248

**Published:** 2006-10-10

**Authors:** Thu Minh T Tran, Hien T Nguyen, Hiroshi Yatsuya, Nobuyuki Hamajima, Akio Nishimura, Katsuki Ito

**Affiliations:** 1 Planning Department, National Institute of Hygiene and Epidemiology, 1 Yersin Hai Ba Trung, Hanoi, Vietnam; 2 Faculty of Public Health, Hanoi Medical University, 1 Ton That Tung Street, Ba Dinh District, Hanoi, Vietnam; 3 Young Leaders' Program, Nagoya University Graduate School of Medicine, 65 Tsurumai-cho, Showa-ku, Nagoya 466-8550, Japan; 4 National Institute of Hygiene and Epidemiology, 1 Yersin Hai Ba Trung, Hanoi, Vietnam; 5 Department of Public Health/Health Information Dynamics, Nagoya University Graduate School of Medicine, 65 Tsurumai-cho, Showa-ku, Nagoya 466-8550, Japan; 6 Department of Preventive Medicine/Biostatistics and Medical Decision Making, Nagoya University Graduate School of Medicine, 65 Tsurumai-cho, Showa-ku, Nagoya 466-8550, Japan; 7 Office of International Cooperation, National Institute of Public Health, 2-3-6 Minami, Wako 351-0197, Japan

## Abstract

**Background:**

Sufficient targeted HIV prevention activities aiming at reducing HIV transmission within and from an extremely marginalized population of injection drug users (IDUs) must urgently and efficiently be implemented in Vietnam. This study was conducted to facilitate the development of such activities by describing transmission risks of young IDUs and evaluating factors in association with HIV infection.

**Methods:**

Thirty clusters were selected from 29 hotspot communes in Long An province by probability proportional to size (PPS) sampling method. The snowball technique was used for enrolling participants in each cluster. The cross-sectional association of factors obtained during direct structured interviews to 248 male IDUs aged 14 to 29 years old and with their HIV test results were examined.

**Results:**

The HIV prevalence among the studied IDUs was 32%. Age range of 18–20 years old, low educational level, sharing injection equipment or injection drug use in the other cities were independently associated with HIV serostatus in the multivariate analysis. Sexual behaviors did not differ between HIV-positive and -negative IDUs. Among HIV seropositive IDUs who had sexual contact with primary (n = 37), casual (n = 6), and commercial (n = 15) partners, only 5.4% (n = 2), 33.3% (n = 2), and 46.7% (n = 7), respectively, responded that they had used condoms every time.

**Conclusion:**

About one-third of young IDUs aged less than 30 identified in the hotspot communes in Long An, Vietnam was found to be infected with HIV, and socio-demographic and injection-related factors might account for the infection risk. Prevailing risky sexual behavior of this extremely marginalized population highlights the need to reduce their high transmission risks as a public health priority.

## Background

The HIV Sentinel Surveillance in Vietnam shows that HIV infection has reached epidemic proportions among injection drug users (IDUs) [1-3]. The observed higher risk of IDUs has been explained by the combination of different risk behaviors such as sharing of syringes and needles, no regular use of condoms, and contact with different sexual partners [4]. Actually, IDUs in general are known to be sexually active [5], and male IDUs who had sexual contacts with female sex workers (FSWs) reportedly have a higher HIV infection risk than IDUs who do not [6,7]. Because the sheer scale of problems involving IDUs and IDU-related HIV infection is far greater in developing and transitional countries [3,8], assessment of the behavioral risks of male IDUs for both themselves and other population including women and children is highly important, and urgently needed especially in Vietnam, where there is little information about this issue compared to other countries located near the Gold Triangle, one of the places producing the largest amount of drugs in the world [9,10]. In addition, Vietnam opened its doors to the outside world in 1988, resulting in an increasing gap between the rich and the poor, which warrants investigations in relation to socio-demographic factors and HIV infection risk. Several studies conducted in Vietnam at different times indicated that HIV infection has been spreading rapidly among drug users who are younger than before [5,11,12], which also highlights the urgent need for prevention activities in young IDUs. This study was conducted to facilitate the development of such targeted HIV prevention activities aiming at reducing HIV transmission within and from IDUs based on harm-reduction strategies. We evaluated the association of socio-demographic factors as well as their injection and sexual behaviors with HIV seroprevalence.

## Methods

### Study area

This is a cross-sectional study conducted from September to November 2002 in Long An Province in Vietnam. Long An is located southwest of Ho Chi Minh City, and north of Cambodia, and has a population of about 1.3 million people [13]. Long An was chosen for the study because of its location and rapid increase in HIV prevalence. The province is also poor and characterized by its highly mobile population, FSWs and IDUs.

### Geographical mapping and probability proportional to size (PPS) sampling

Male IDUs, defined as men who used non-medically prescribed injection drugs a month previous to the survey, were included in the study no matter how often they had injected. There were no exclusion criteria. We confirmed whether the participant met this criterion at the time of interview. We did not include female IDUs in the present study because in-depth interviews earlier revealed them to be few in the province. We first mapped IDUs geographically based on the information from the survey supporters such as peer trainers of IDUs or FSWs, and officials from the Women's or Youth Union or police station. From 71 communes originally included in Long An Province, we selected 29 hotspot communes that had a high density of IDUs, drug dealers and mobile population, places that could be considered hazardous for HIV transmission. They are located along main roads and have borders with Ho Chi Minh City or Cambodia. An additional purpose of selecting these hotspot communes was for subsequent interventions. Thus, the target population of the present analysis can be described as IDUs who were identified in these hotspot communes, and they may not be representative of the whole IDU population in the province. Using a probability proportional to size (PPS) cluster sampling method, 30 clusters from 29 hotspot communes where 791 IDUs had been mapped were randomly selected. Only clusters in a commune that included at least 15 IDUs were eligible to be sampled. These procedures were carried out by the investigators who had been selected from local health staffs, and had attended a three-day training course provided by study supervisors from the Faculty of Public Health in Hanoi Medical University. The mapping process and sampling procedures followed the behavioral surveillance surveys guideline published by Family Health International[14].

### Participants

The aforementioned investigators conducted face to face interviews with the assistance of survey supporters. These investigators (interviewers) were trained not only for the interview skills but also regarding the psychological aspect of IDUs to gain their full trust in order to have them talk honestly and then provide effective counseling. As mentioned above, interviewers were local health staff who had a certain level of knowledge and skills, and health care workers in Vietnam are generally trusted by the high risk populations because of the care and support they often provide. In each cluster, ten IDUs were interviewed using the snowball technique. Briefly, the first interviewee was randomly selected by the survey supporters at each location. After the interview, the interviewer asked the interviewee to introduce or invite another participant from among the IDUs he knew. The interviews continued until ten IDUs were enrolled. As described later, participants were also asked for HIV testing after his interview although agreement to HIV testing was not among the eligibility criteria. Interviewees were informed verbally about the objectives of the study, and that the result would be kept strictly confidential. They were also provided written information that briefly described the objective and content of the study, and the contact information with their identification numbers, which could be used to request their HIV test results by phone. In total, 300 IDUs were interviewed and 284 had their blood tested in the present survey. Although the ages of the contacted IDUs ranged from 14 to 49 years old, we excluded 36 subjects (13%) who were 30 years old or more, leaving 248 subjects eligible for the present analysis. Participation was solely on a voluntary basis, and measures were taken to assure confidentiality. The study protocol and informed consent procedure were approved by the Hanoi Medical University Ethics Committee.

### Interview process and pre- and post-test counseling

Interview and collection of blood sample were conducted wherever IDUs felt comfortable, e.g., in a corner of a park, on the street, in the place where they used injection drugs, in Karaoke bar, and in a friend's house etc. If the participants agreed to take blood testing for HIV invited after the interview, a trained laboratory technician performed the blood draw. In any cases, the interviewers gave pre-test counseling explaining the importance of knowing their HIV serostatus, emphatically assured them as to the confidentiality of the information, and thus encouraged the participants to obtain their test results. Out of the 300 IDUs interviewed, 284 agreed to take HIV blood tests. Among the 284 IDUs blood tested regardless of age, 227 (80%) received their test results. The staff did not inform them of their test results without providing counseling. Post-test counseling was given when participants called in for the test results. IDUs with HIV negative test result received counseling by telephone or at the office. IDUs with positive test results were asked to come to the office to obtain their results with complete post-test counseling.

### Variables

The interview was carried out using a questionnaire comprised of several parts including the socio-demographic characteristics of IDUs as well as the behaviors related to injection and sexual contacts. The methods used to obtain information are described in the following.

#### Socio-demographic factors

Age at the time of the survey was calculated by subtracting the year of birth from 2002, and the participants were categorized into those aged 14–17, 18–20, 21–24, and 25–29 years old. Educational level was assessed in five categories; illiteracy, primary (1–5 years), secondary (6–9 years), high school (10–12 years), and college or university. It was grouped in the analysis into three categories: illiteracy/primary, secondary, and high school or more. Marital status was assessed in five categories; never married, currently married, divorced, separated (divorce not officially registered), and widowed. No one was widowed, and only 5 were divorced or separated. We combined categories other than currently married in the analysis into currently not married.

#### Factors related to injection

The year when an interviewee started using injection drugs was assessed. Their initiation age was categorized into 4 groups (10–17, 18–20, 21–24, and 25–29 years old) for the analyses. The duration of their injection drug use was calculated and categorized into 3 groups: less than 1 year, from 1 to 2 years, and 3 years or more. Their injection frequency in the previous month was assessed and grouped into two categories (once a day or more or others). Subjects were asked about the frequency of sharing the injection equipment by the question, "In the last month have you used a syringe and needle that others had or have just used?" The answer was assessed in 5 categories: never, sometimes, about half, almost every time, and every time. Because of the small number of participants had responded almost every time (n = 3) or every time (n = 2), these two categories were combined. Participants were also asked whether or not they had injected in other cities in the previous year.

#### Factors related to sexual behavior

The number of persons with whom the IDUs had sexual contacts in the previous year was inquired according to 5 categories (none, 1, 2, 3–4, and 5 or more). Types of sex partners were also surveyed in terms of wife/girlfriend, casual partners, or FSWs (multiple answers were allowed). Casual partners in this study were defined as persons who were neither girlfriend or wife, nor female sex workers, but rather someone who could not be described as a main and serious partner. Whether or not they had used condoms with each type of sex partners was assessed by the question, "How often did you use condoms during sexual contacts with wife/girlfriend (casual partners, or FSWs) in the last 12 months?" The possible answers were: never, sometimes, about half, almost every time, and every time.

### Laboratory method

The blood test was conducted in a laboratory at the Provincial Center for Preventive Medicine using Testing Strategy III from the Viet Nam Protocol of HIV testing, which included one quick test and two ELISA Screening tests to confirm HIV seropositivity. Those with positive results in all the three tests were considered as HIV seropositive.

### Data management and statistical analysis

Data were entered by two different people using Epi-Info version 6.04d (CDC, Atlanta, GA), then analyzed with SPSS version 11.5J (SPSS, Chicago, Illinois, USA). Differences in proportions between HIV positive and negative IDUs were tested by χ^2 ^tests. Continuous variables were compared by Student *t*-tests. We employed bivariate and multivariate logistic regression analyses with HIV serostatus as a dependent variable in order to evaluate the role of socio-demographic and injection-related factors while simultaneously controlling for possible confounding effects of age or other factors. The model was not constructed for predicting individual estimates of risk. Variables related to sexual behaviors were not examined by logistic regression analyses. Multivariate logistic regression analysis included all the variables that were significantly (P < 0.05) or nearly significantly (P < 0.10) associated with HIV in age-category-adjusted bivariate analyses with the forced variables entry method. Bivariate logistic regression against the injection initiation age was not performed to avoid collinearity that might have occurred if age had been adjusted due to a strong correlation between the ages of the IDUs at the time of the survey and when they started injection (Pearson's *r *= 0.75, P < 0.001). Adjusted odds ratios (ORs) and their 95% confidence intervals were presented.

## Results

The prevalence of the HIV infection was 80 (32%) among the 248 IDUs aged 29 years old or less. The mean age of IDUs was 21.3 (standard deviation [SD]: 3.1, range: 14–29) years. The mean ages of HIV positive and negative IDUs were 21.1 and 21.3, respectively (P = 0.56, Student *t*-test). The proportion of the IDUs aged 17 years or younger, 18 to 20 years, 21 to 24 years and 25 to 29 years was 11.3%, 33.5%, 38.3%, and 16.9%, respectively. Most of the subjects (98.8%) were living with their relatives or family at the time of the survey. A high school or higher educational level was associated with lower prevalence of HIV (age-adjusted odd ratio (OR): 0.69, P = 0.034).

In the present study sample, 69.8% of IDUs started their injection drug use in the year 2000 or later (Table 2). The mean ages when IDUs began using drugs or injection drugs were 19.0 and 19.4 years old, respectively. The proportions of IDUs who started injection drugs at ages 10–17, 18–20, 21–24, and 25–29 were 27.4%, 41.5%, 24.2%, and 6.8%, respectively. The IDUs mainly used heroin (95.2%), but some used opium (10.9%) including 20 subjects (8.1%) that used both drugs. The proportions of IDUs using injection drugs less than 1 year, 1 to 2 years, and 3 years or more were 22.1%, 48.0%, and 29.8%, respectively. Once a day or more use of injection drugs, sometimes sharing of injection equipment or having used injection drugs in the other cities were associated with HIV seropositivity (age-adjusted OR: 1.70, 2.05 and 1.82, respectively). Multivariate logistic regression analysis revealed that 18–20 years of age compared to the 14–17 group (OR: 3.30, P = 0.034), and illiteracy/primary and secondly educational level compared to high school or more (OR: 2.37 and 2.07, P = 0.045 and 0.044, respectively) were significantly associated with HIV serostatus (Table 3). Injection drug use in the other cities (OR: 1.69, P = 0.089) and sometimes sharing injection equipments with others (OR: 1.91, P = 0.082) were also associated with HIV serostatus with borderline significance.

The proportions of IDUs with multiple (2 or more) sexual partners were 41.7% (Table 4). There was no significant association between the number of sexual partners and HIV seropositivity, but at least 20 out of the 80 HIV seropositive IDUs (25.0%) had multiple sex partners. The proportion of IDUs who had sexual contacts with FSWs was 30.0%. More than half (54.9%) of the IDUs who had sexual contacts with FSWs did not use condoms every time (Table 5). About one quarter of those who did not use condoms every time in their contacts with FSWs (28.6%, 8 out of 28) were HIV seropositive. Nearly all (94.1%) the IDUs reported that they did not use condoms every time in their contacts with their wives/girlfriends. Thirty-five out of 111 IDUs (31.5%) who did not use condoms every time with their wives/girlfriend were HIV seropositive.

Among 80 HIV seropositive IDUs, 5.0% (n = 4) and 22.5% (n = 18) had shared needles or syringes more than half the time, or at least sometimes, respectively in the previous month of the survey. Two out of four IDUs (50.0%) who had shared needles more than half times had never used condoms with their wife or girlfriend. Ten out of 18 IDUs (55.6%) who had sometimes shared needles had never used condoms with their wife or girlfriend. Similar, but less pronounced trend was observed among HIV negative IDUs (33.3% and 45.5%, respectively).

## Discussion

The major findings of the present study are that the prevalence of HIV among a relatively young IDU sample enrolled in the hotspot communes in Long An, Vietnam is 32%, and that 18–20 years of age, low educational level, sharing injection equipment or injection drug use in the other cities were independently associated with HIV serostatus [15]. More than 1 out of 4 HIV infected IDUs were found to have shared needles in the previous month of the survey. About half of the IDUs who had shared needles never used condoms at their sexual contacts with primary partners regardless of the HIV serostatus. These results first suggest that current measures have not been sufficient to reduce risk behaviors in Vietnam, and that more efforts to reduce transmission risks of HIV infected IDUs would be needed.

Males aged 18 to 20 years old had a significantly higher HIV seroprevalence in the IDUs, and the association was independent of other factors. The result may be consistent with the previous studies conducted among IDUs and FSWs in Vietnam [1,16], although the phenomenon may have been caused by the dropout due to dying or severe AIDS, which is less frequent among the younger IDUs or among those who started drug use more recently. Younger age is generally related to a lower economic status [10,17,18], which might have predisposed young IDUs to risky behaviors such as unsafe sex or sharing the injecting equipment, although age and these risk behaviors were not significantly associated in the present analysis. Because we sampled IDUs in the hotspot communes using the snowball method, they may not be representative of the whole IDU population in the province. Their socio-economic characteristics as well as the injection and sexual behaviors might have been similar reducing inter-individual variability in the variables. Although those aged 18 to 20 years seemed to be associated with higher HIV prevalence in the present sample, generalizability of the finding would be limited. Further studies are needed for better understanding of the risks associated with IDUs' age on HIV infection.

The duration of injection drug use or the age when beginning injection drugs was not associated with HIV serostatus in the present study, although it would reflect cumulative injection risk for HIV infection [4,19,20]. On the contrary, those with longer than 1 year but not exceeding 3 years use of injection drugs had a higher prevalence of HIV compared to those with less than 1 year of use. Although the failure of serologic assays during the window period in individuals shortly after infection might be associated with the lower seroprevalence in the reference group [21], it remains unclear why the IDUs' use of injection drugs for 3 years or more was not associated with increased prevalence. It could be speculated that the HIV-infected IDUs with a relatively longer history of drug injection might not be included in the present sample because their general health conditions were poor due to progression of the HIV infection [22]. Again, the present sample are not representative of the whole IDU population, so further studies using other sampling methods or with prospective design are needed to analyze the relation between the duration of injection drug use and HIV.

The adoption of injection reportedly occurred within a year on average [23], and a short transition period was reported to be an HIV risk [24]. We also found that the mean ages of IDUs when they started using drugs were only slightly lower than when they became injectors (19.0 vs. 19.4 years old, respectively), and most of the IDUs adopted the injection habit within a year (73.0%) or one to two years (17.3%). But we did not find any association between the duration of the transition period and HIV seroprevalence (data not shown).

The sexual behaviors of the IDUs reported at the time of the survey were not associated with their HIV serostatus. And, although not statistically significant, the association of FSWs and HIV infection seems rather contradictory [16]. Because the population of the present study does not represent the variability in the studied variables which would have existed if the whole IDU population had been sampled, no firm speculation based on the statistical testing could be made. Nevertheless, it may be possible that the IDUs who did not have sexual contacts with FSWs were of a lower economic status that also predisposed them to a much riskier situation [25,26], or that HIV infection might have been the cause, rather than the effect of, not having contacts with FSWs [26,27]. From another perspective, more than one-third of the IDUs responded that they had had multiple sex partners including FSWs, and that condoms had not been regularly used irrespective of their HIV serostatus. These cross-sectional results indicated that the heterosexual intercourse of the young IDUs in Vietnam would not be so much a source of their HIV infection [28] as a transmission risk, or a cause of spread to other populations [5,9,25,29,30]. They could well have served as a bridge in the transmission of HIV to other populations such as FSWs, clients of FSWs, wives of clients, children, and to the general population [11,31]. Actually, these modes of transmission have been frequently observed in countries that experienced the transition from a concentrated to a generalized epidemic [32]. Although Vietnam is considered as a concentrated epidemic country [2], the fact that 32% of IDUs were found to be infected with HIV emphasized the importance of the present study which described and evaluated socio-demographic characteristics of IDUs, as well as the prevailing hazardous injection and sexual behaviors. HIV prevention efforts targeting IDUs must not underestimate the role of sexual transmission in the spread of infection among and from IDUs [10,33]. More specifically in order to reduce sexual risks among IDUs, continuous efforts should be made to increase awareness not only of IDUs but also of others including HIV/AIDS staff or health care workers about the fact that IDUs are at high risk of HIV infection and transmission by unsafe sex as well as by sharing needles. Emphasis should be placed on the fact that many IDUs are sexually active and their primary sexual partners are at high infection risk. All information, education, and communication activities and behavior change interventions should include condom promotion component, and condom distribution/promotion activities can also be integrated to exchange/provision campaign of clean needles and syringes. The present results should be used to enhance or modify the development of such measures as to monitor risk behaviors in hidden populations and to improve preventive strategies to reduce transmission risk as a public health priority not only in Vietnam but in other countries as well.

Having used injection drugs in the other cities was associated with HIV seropositivity in the age-adjusted model. Among those who had used injection drugs in other cities, 91.3% migrated to Ho Chi Minh City, where the prevalence of IDU is the highest in Vietnam (73.6%) according to the report from the AIDS Division, Ministry of Health, Vietnam. It could be that those mobile IDUs had spread HIV infection from city to city. Epidemiologically, migrants have been known to be vulnerable to HIV risk [24,34]. Riskier sexual behaviors were reported in traveling individuals with HIV [35]. Migration is often related to IDUs socioeconomic status (SES), and economic distress may have affected the IDUs who gave an affirmative answer to this question. Such conditions usually accompany a situation with limited access to appropriate information on HIV. Moreover, mobile IDUs may have visited shooting galleries where transmission risk of HIV would be high [36]. Less availability of social support was also reported in migrant IDUs, and was presumably associated with their risk-taking behaviors [37]. At the same time, there may be sexual or addiction-related motives that have driven them to cities where a certain social network exists despite their knowledge of the risks involved. It is also possible that HIV-infected IDUs tended to move to other cities to use injection drugs [10].

Further investigations are warranted with more detailed assessment of IDUs' geographic movement in relation to their sexual and injection behaviors so as to disentangle why this factor was related to IDUs' own HIV infection risk. In-depth qualitative studies on FSWs are also urgently needed since they often use injection drugs, are highly mobile, and are more vulnerable to HIV due to the lack of power or supportive environment, or peer pressure in a new area. Better understanding of these issues would help us to speculate or model how injection and sexual risk among IDUs may contribute to the larger epidemic not only within Vietnam but in the region located near Gold Triangle as a whole.

As with several other studies of IDUs[9,11,12], the limitations of the present study should be kept in mind. First, the present results were based on self-reports that might have been affected by ideas about social desirability or denial of recent risk behaviors. About one-third of IDUs (n = 80), all of whom identified themselves as never married, did not answer to the questions dealing with their sexual behavior. Although we obtained similar results from sensitivity analyses conducted as if these people had had either no or five or more sexual partners, caution is needed due to possible misclassification of the variable. In addition, we did not include any inquiries related to concurrency in partnership or homosexuality because we have paid less attention to the latter issue in Vietnam [38]. It is imaginable that disclosure of this issue may also be limited, and thus we have probably been underestimating the actual situation. Further investigations with an appropriate assessment of these issues are needed, possibly using some device to guarantee complete privacy. In terms of drug use-related inquiries, individual and contextual level factors related to injection initiation, chronological change in the level or pattern of drug use together with that of sexual activities, and characteristics of the drug use network should be assessed in future studies from both epidemiological and preventive points of view [39]. The number of needles exchanged in the area could be used as a surrogate measure of needle sharing. Since the information would make respondents aware that they should not share injection equipment or have sex without condoms, they may have tended to give a socially desirable response. Although this has serious implications when inferring causal relationships among them, the transmission risks evaluated in the present study underscore the urgent need for intervention as a public health priority.

Second, we did not obtain detailed information on the participant SES, nor did we inquire as to the reason why the IDUs had gone to other cities and had used injection drugs there. As mentioned in the earlier discussion of this issue, SES, both at the individual, family or community level, would have had a significant impact on the behavior risks, and future study must obtain a valid measure of SES in order to contribute to the development of effectively targeted prevention activities.

In future studies, not just general knowledge about HIV/AIDS of the IDUs but what has been causing barriers for behavior modification should be investigated. Health beliefs of IDUs should also be evaluated. Other psychiatric disorders including depression, post-traumatic disorders, and childhood abuse should be associated with their risk behaviors. In addition, other quantifiable assessments of drug use such as blood or urine test may well be combined.

Finally, it is usually not possible to determine the chronology between the associated factors and the actual HIV infection. Subjects who knew they were HIV seropositive might have changed to safe behaviors. Hence, the results of this study should be interpreted with caution and with respect for these limitations. Ideally, prospective observation or interventional studies should also be conducted.

## Conclusion

In summary, being 18 to 20 years old, low educational level, sharing injection equipment or injection drug use in the other cities were associated with HIV seroprevalence among the studied IDUs in the hotspot communes in Long An, Vietnam. Prevailing risky sexual behavior of this extremely marginalized population highlights the need to reduce their high transmission risks as a public health priority. These findings also indicate the importance of addressing these issues including behavior education, supplying clean needles and syringes, condom distribution, and encouragement of HIV testing [7,40,41] as an efficient way to reach out to these hidden populations.

## Competing interests

The author(s) declare that they have no competing interests.

## Authors' contributions

HTN developed the concept and design of the study, and supervised the implementation of the project and data collection. TMTT and HY analyzed the data and wrote the paper. NH, AN, and KI participated in interpretation of the data and revision of the paper. All authors read and approved the final manuscript.

## Pre-publication history

The pre-publication history for this paper can be accessed here:



## Figures and Tables

**Table 1:** Socio-demographic characterestics of injection drug users according to HIV serostatus, and odds ratios associated with HIV seropositivity (n = 248)

**Characteristics**	**HIV (+) (n = 80)**	**HIV (-) (n = 168)**	p-value* (χ^2 ^test)	**OR^†^**	**95%CI**	**p-value**
**Age (years)**						
14–17	5 (6.3)	23 (13.7)		1	(reference)	
18–20	32 (40.0)	51 (30.4)	0.22	2.89	0.99 – 8.36	0.051
21–24	31 (38.8)	64 (38.1)		2.23	0.77 – 6.42	0.14
25–29	12 (15.0)	30 (17.9)		1.84	0.57 – 5.97	0.31
**Educational level**						
High school (10–12 years) or more	15 (18.8)	52 (31.0)		1	(reference)	
Secondary (6–9 years)	45 (56.3)	84 (50.0)	0.12	1.97	0.99 – 3.93	0.053
Illiteracy/primary (1–5 years)	20 (25.0)	32 (19.0)		2.42	1.07 – 5.50	0.035
**Marital status**						
Currently married	14 (17.5)	23 (13.7)	0.45	1	(reference)	
Currently not married^‡^	66 (82.5)	145 (86.3)		0.69	0.32 – 1.49	0.34

n indicates number; OR, odds ratio; CI, confidence interval.

*: Differences in proportions between HIV (+) IDUs and HIV (-) IDUs were tested by χ^2 ^tests.

†: Age-category-adjusted odds ratio for the characteristics except 'Age'.

‡: Currently not married category includes never married (n = 206) and divorced/separated (n = 5).

**Table 2:** Characteristics of subjects related to drug use according to HIV serostatus, and odds ratios associated with HIV seropositivity (n = 248)

**Characteristics**	**HIV (+) (n = 80)**	**HIV (-) (n = 168)**	p-value*(χ^2 ^test)	**OR^†^**	**95%CI**	**p-value**
**Year they started injection drug**						
-1999	20 (25.0)	55 (32.7)	0.22	1	(reference)	
2000-	60 (75.0)	113 (67.3)		1.56	0.83 – 2.91	0.17
**Age when they started injection drug (years)**						
10–17	24 (30.0)	44 (26.2)				
18–20	36 (45.0)	67 (39.9)	0.23	NA^‡^		
21–24	13 (16.3)	47 (28.0)		NA		
25–29	7 (8.8)	10 (6.0)		NA		
**Duration of injection drug use (years)**						
< 1	14 (17.5)	41 (24.4)		1	(reference)	
1 to 2	46 (57.5)	73 (43.5)	0.12	1.83	0.87 – 3.84	0.12
3-	20 (25.0)	54 (32.1)		1.05	0.45 – 2.44	0.11
**Frequency of drug use (previous month)**						
<1/day	31 (38.8)	87 (51.8)	0.055	1	(reference)	
≥1/day	49 (61.3)	81 (48.2)		1.70	0.98 – 2.96	0.058
**Sharing needles and syringes (previous month)**						
Never	58 (72.5)	140 (83.3)		1	(reference)	
Sometimes	18 (22.5)	22 (13.1)	0.26	2.05	0.99 – 4.08	0.054
About half	2 (2.5)	3 (1.8)		1.75	0.27 – 10.88	0.57
Every time/Almost every time	2 (2.5)	3 (1.8)		1.66	0.26 – 10.29	0.61
**Injection drug use in other cities (12 months)**						
No	51 (63.8)	126 (75.0)	0.067	1	(reference)	
Yes	29 (36.3)	42 (25.0)		1.82	1.01 – 3.26	0.046

n indicates number; OR, odds ratio; CI, confidence interval; IDU, injection drug user.

*: Differences in proportions between HIV (+) IDUs and HIV (-) IDUs were tested by χ^2 ^tests.

†: Age-category-adjusted odds ratio.

‡: Not applicable due to strong correlation between ages at time of survey and when they started injection drug use.

**Table 3:** Results of multivariate logistic regression analyses against HIV seropositivity with socio-demographic and injection characteristics of IDUs

	**n**	**OR**	**95%CI**	**p-value**
**Age (years)**				
14–17	28	1	(reference)	
18–20	83	3.30	1.09 – 9.94	0.034
21–24	95	2.46	0.81 – 7.41	0.11
25–29	42	1.54	0.45 – 5.34	0.49
**Educational level**				
High school (10–12 years) or more	67	1	(reference)	
Secondary (6–9 years)	129	2.07	1.02 – 4.21	0.044
Illiteracy/primary (1–5 years)	52	2.37	1.02 – 5.51	0.045
**Frequency of drug use (previous month)**				
<1/day	118	1	(reference)	
≥1/day	130	1.60	0.90 – 2.84	0.11
**Injection drug use in other cities (12 months)**				
no	177	1	(reference)	
yes	71	1.69	0.92 – 3.11	0.089
**Sharing needles and syringes (previous month)**				
Never	198	1	(reference)	
Sometimes	40	1.91	0.92 – 3.96	0.082
About half	5	1.23	0.18 – 8.49	0.83
Every time/Almost every time	5	1.32	0.19 – 9.08	0.77

n indicates number; OR, odds ratio; CI, confidence interval; IDU, injection drug user.

*: Multivariate logistic models include age-category, educational level, frequency of injection drug use, injection drug use in other city, and sharing injection equipment as independent variables.

**Table 4:** Characteristics of subjects related to sexual behaviors according to HIV serostatus (n = 248)

**Characteristics**	**HIV (+) (n = 80)**	**HIV (-) (n = 168)**	**Total**	p-value*(χ^2 ^test)
**Number of persons with whom IDUs had sexual contacts in previous year**				
None	9 (11.3)	17 (10.1)	26 (10.5)	
1	28 (35.0)	44 (26.2)	72 (29.0)	
2	8 (10.0)	19 (11.3)	27 (10.9)	0.77
3–4	5 (6.3)	15 (8.9)	20 (8.1)	
5-	7 (8.8)	16 (9.5)	23 (9.3)	
No answer	23 (28.8)	57 (33.9)	80 (32.3)	
**Sexual contacts with wife/girlfriend in previous year (total response: n = 168)**				
Yes	37 (64.9)	80 (72.1)	117 (69.6)	0.34
No	20 (35.1)	31 (27.9)	51 (30.4)	
**Sexual contacts with casual partners in previous year (total response: n = 168)**				
Yes	7 (12.3)	12 (10.8)	19 (11.3)	0.78
No	50 (87.7)	99 (89.2)	149 (88.7)	
**Sexual contacts with FSWs in previous year (total response: n = 168)**				
Yes	13 (22.8)	37 (33.3)	50 (29.8)	0.16
No	44 (77.2)	74 (66.7)	118 (70.2)	

n indicates number; IDU, injection drug user.

*: Differences in proportions between HIV (+) IDUs and HIV (-) IDUs were tested by χ^2 ^tests.

**Table 5:** Use of condoms with three types of sex partners in the previous year according to HIV serostatus

**Use of condoms with**	**HIV (+)**	**HIV (-)**	**Total**	p-value* (χ^2 ^test)
**Wife or girlfriend (valid response: n = 118)**				
Every time	2 (5.4)	5 (6.2)	7 (5.9)	
Almost every time	1 (2.7)	3 (3.7)	4 (3.4)	
About half	2 (5.4)	3 (3.7)	5 (4.2)	0.91
Sometimes	3 (8.1)	11 (13.6)	14 (11.9)	
Never	29 (78.4)	59 (72.8)	88 (74.6)	
**Casual partners (valid response: n = 19)**				
Every time	2 (33.3)	4 (30.8)	6 (31.6)	
Almost every time	0 (0.0)	1 (7.7)	1 (5.3)	
About half	0 (0.0)	0 (0.0)	0 (0.0)	0.85
Sometimes	1 (16.7)	1 (7.7)	2 (10.5)	
Never	3 (50.0)	7 (53.8)	10 (52.6)	
**FSWs (valid response: n = 51)**				
Every time	7 (46.7)	16 (44.4)	23 (45.1)	
Almost every time	1 (6.7)	10 (27.8)	11 (21.6)	
About half	1 (6.7)	0 (0.0)	1 (2.0)	0.28
Sometimes	3 (20.0)	5 (13.9)	8 (15.7)	
Never	3 (20.0)	5 (13.9)	8 (15.7)	

n indicates number; IDU, injection drug user.

*: Differences in proportions between HIV (+) IDUs and HIV (-) IDUs were tested by χ^2 ^tests.
